# Extended T2-IVIM model for correction of TE dependence of pseudo-diffusion volume fraction in clinical diffusion-weighted magnetic resonance imaging

**DOI:** 10.1088/1361-6560/61/24/N667

**Published:** 2016-11-28

**Authors:** N P Jerome, J A d’Arcy, T Feiweier, D-M Koh, M O Leach, D J Collins, M R Orton

**Affiliations:** 1 Cancer Research UK Cancer Imaging Centre, Division of Radiotherapy & Imaging, The Institute of Cancer Research, London, SM2 5NG, UK; 2 Siemens Healthcare, Erlangen, Germany; 3 Department of Radiology, Royal Marsden Hospital, Sutton, Surrey, UK; pmbaa43em1 martin.leach@icr.ac.uk

**Keywords:** diffusion-weighted magnetic resonance imaging, functional magnetic resonance imaging, intravoxel incoherent motion, reproducibility of results, IVIM, T2IVIM, perfusion magnetic resonance imaging

## Abstract

The bi-exponential intravoxel-incoherent-motion (IVIM) model for diffusion-weighted MRI (DWI) fails to account for differential *T*_2_*s* in the model compartments, resulting in overestimation of pseudodiffusion fraction *f*. An extended model, T2-IVIM, allows removal of the confounding echo-time (TE) dependence of *f*, and provides direct compartment *T*_2_ estimates. Two consented healthy volunteer cohorts (*n*  =  5, 6) underwent DWI comprising multiple TE/*b*-value combinations (Protocol 1: TE  =  62–102 ms, *b*  =  0–250 mm^−2^s, 30 combinations. Protocol 2: 8 *b*-values 0–800 mm^−2^s at TE  =  62 ms, with 3 additional *b*-values 0–50 mm^−2^s at TE  =  80, 100 ms; scanned twice). Data from liver ROIs were fitted with IVIM at individual TEs, and with the T2-IVIM model using all data. Repeat-measures coefficients of variation were assessed for Protocol 2. Conventional IVIM modelling at individual TEs (Protocol 1) demonstrated apparent *f* increasing with longer TE: 22.4  ±  7% (TE  =  62 ms) to 30.7  ±  11% (TE  =  102 ms); T2-IVIM model fitting accounted for all data variation. Fitting of Protocol 2 data using T2-IVIM yielded reduced *f* estimates (IVIM: 27.9  ±  6%, T2-IVIM: 18.3  ±  7%), as well as *T*_2_  =  42.1  ±  7 ms, 77.6  ±  30 ms for true and pseudodiffusion compartments, respectively. A reduced Protocol 2 dataset yielded comparable results in a clinical time frame (11 min). The confounding dependence of IVIM *f* on TE can be accounted for using additional *b*/TE images and the extended T2-IVIM model.

**Abbreviations**
ADC: Apparent diffusion coefficient
CoV: Coefficient of variation
DWI: Diffusion weighted imaging
EPI: Echo-planar imaging
GRAPPA: Generalised autocalibrating partially parallel acquisition acceleration
IVIM: Intravoxel incoherent motion
MR: Magnetic resonance
ROI: Region of interest
SNR: Signal-to-noise ratio
SPAIR: Spectral adiabatic inversion recovery
TE: Echo time
TR: Repetition time

## 1. Introduction

Diffusion-weighted imaging (DWI) is an important functional imaging technique in oncology, where signal intensity modulated by the (diffusive) motion of water molecules can be used to inform on tumour cellularity, tortuosity of extracellular space, and microstructural organisation. While the apparent diffusion coefficient (ADC), conventionally derived by a two-point measurement with application of diffusion-sensitising magnetic field gradients of varying strengths (*b*-values), has shown utility in oncology for disease localisation, diagnosis, staging and assessing therapy response (Yamada *et al* 1999, Taouli *et al* 2009, Rosenkrantz *et al* 2010, Lee *et al* 2011, Pope *et al* 2012, Song *et al* 2013), the diffusion decay curve in tissues is often observed to deviate from the single exponential behaviour expected by simple Gaussian diffusion (Lemke *et al* 2009, Koh *et al* 2011, Rosenkrantz *et al* 2015, Winfield *et al* 2015, Jerome *et al* 2016). The nature and utility of this non-monoexponential signal decay observed in multiple *b*-value DWI is a source of much discussion (Chandarana *et al* 2011, Dyvorne *et al* 2013). The two-compartment intra-voxel incoherent motion (IVIM) model proposed by Le Bihan *et al* (1988) is a popular choice for diffusion studies in the body, with the associated pseudo-diffusion volume parameter *f* being a potentially useful biomarker in oncology for lesion characterisation or response. In the two-compartment model framework, components are commonly taken to represent pseudo-diffusion and true diffusion, which may in turn represent vascular and tissue compartments, giving signal dependence on *b*-value according to:

1 \begin{eqnarray*}S\left(b,\text{TE}\right)={{S}_{0}}\exp \left(-\text{TE}/{{T}_{\text{2}}}\right)\left[f\exp \left(-b{{D}^{\ast}}\right)+\left(\text{1}\,-\,f\right)\exp \left(-bD\right)\right]\end{eqnarray*}

Where *f* is the pseudo-diffusion volume fraction, *D* and D^*^ are the true- and pseudo-diffusion coefficients, and *T*_2_ is the transverse relaxation time, implicitly assumed to be the same in both compartments. Standard DWI protocols are commonly acquired at a single echo time (TE), most often the minimum, determined by the time required to include the largest diffusion gradient alongside the spin-refocusing pulse and the imaging readout. The TE and *T*_2_ dependency is typically absorbed into the signal scaling term *S*_0_.

It is known, however, that blood and tissue have distinct and variable *T*_2_ values, and failure to account for this in the IVIM model leads to the mis-assignment of differential *T*_2_ signal decay between compartments, and thus incorrect estimation of the relative fractional volumes of the two compartments. Specifically, as blood is known to have a longer *T*_2_ than tissue, the pseudo-diffusion volume fraction *f* is overestimated as a function of increasing echo time (Lemke *et al* 2010). The early IVIM literature recognises the potential for this assumption to interfere with the IVIM modelling of DWI: ‘…the perfusion factor is obtained, depending on the difference in *T*_2_ between the static and flowing component’ (Le Bihan *et al* 1988).

Inclusion of distinct transverse relaxation constants, referred to in this work to as *T*_2*p*_ and *T*_2*t*_ for pseudo- and true diffusion compartments respectively, modifies the standard IVIM model for echo time dependency (equation (2)):

2 \begin{eqnarray*}S\left(b,\text{TE}\right)={{S}_{0}}\left[f\exp \left(-b{{D}^{\ast}}\right)\exp \left(-\text{TE}/{{T}_{\text{2}p}}\right)+\left(\text{1} ~ -\,f\right)\exp \left(-bD\right)\exp \left(-\text{TE}/{{T}_{\text{2}t}}\right)\right]\end{eqnarray*}

where *S*_0_ is a scaling term independent of both diffusion and *T*_2_ effects, and it is implicitly assumed that repetition time (TR) is long enough to ensure no significant modulation of the signal from incomplete *T*_1_ relaxation. An *apparent* pseudo-diffusion volume fraction can be defined using equation (2) by taking a weighted combination of the terms scaling the *b*-value dependent exponentials, that is:

3 \begin{eqnarray*}{{f}_{\text{app}}}\left(\text{TE}\right)=f\exp \left(-\text{TE}/{{T}_{\text{2}p}}\right){{\left[f\exp \left(-\text{TE}/{{T}_{\text{2}p}}\right)+\left(\text{1}\,-\,f\right)\exp \left(-\text{TE}/{{T}_{\text{2}t}}\right)\right]}^{-\text{1}}}\end{eqnarray*}

This formula gives the pseudo-diffusion volume fraction that would be estimated using equation (1) for a given set of parameters and echo time. Thus, when *T*_2*p*_  =  *T*_2*t*_ we have *f*_app_(TE)  =  *f*, which does not depend on TE, but whenever *T*_2*p*_  ≠  *T*_2*t*_ the pseudo-diffusion volume fraction estimated using equation (1) will depend on TE to some degree.

While it would be desirable to acquire a full sampling of the *b*-value/TE space for fitting of the extended T2-IVIM model in equation (2) to obtain both diffusion and relaxation parameters for each compartment, this is limited in practice by available signal-to-noise ratio (SNR) at larger *b*-TE combinations, and the potential for the acquisition time to become prohibitively long for a clinical examination.

The purpose of this prospective volunteer study is to develop and present a clinically feasible multiple *b*-TE measurement, enabling estimation of the pseudo-diffusion volume fraction in the liver that is not dependent on the echo time. In doing this, we demonstrate that the *T*_2_ relaxation times of both compartments are distinct and can be estimated directly from the data, and which are thus available as biomarkers in their own right, in contrast to previous methods that take an assumed value for *T*_2_ associated with the pseudo-diffusion compartment (Lemke *et al* 2010).

## 2. Materials and methods

### 2.1. MRI acquisition

**Figure 1.**
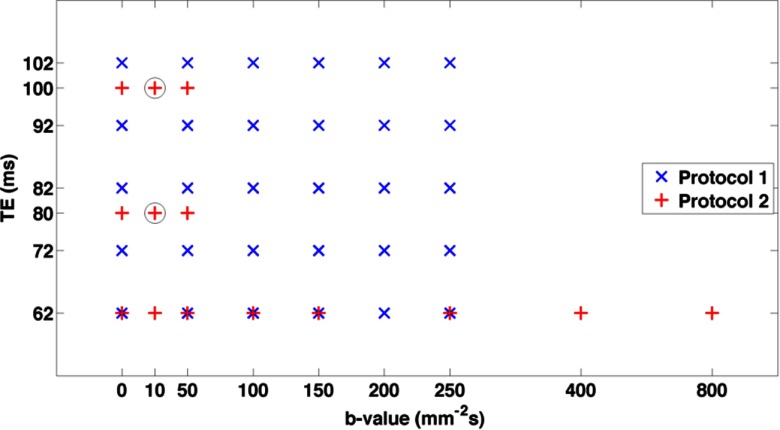
Schematic of acquisition strategy, showing *b*-TE value combinations in Protocol 1 (blue, 5 signal averages) and 2 (red, 12 signal averages). Circles indicate excluded data for the ‘reduced’ subset of Protocol 2 (6 signal averages, making the clinically-practical 11 min acquisition).

This prospective study was approved by the Institutional Review Board. Eleven volunteers were consented (age range 25–61, median 31) and imaged in two groups using two protocol variants with different *b*-values and echo times. Protocol 1 used five volunteers and acquired all combinations of six *b*-values: 0, 50, 100, 150, 200, 250 mm^−2^s and five TEs: 62, 72, 82, 92, 102 ms in order to explore the effect of TE on the signal curve. The ranges of *b*-values and TEs were selected to explore the *b*-TE space while retaining adequate signal-to-noise ratio (SNR); the range of 0–250 mm^−2^s means the data are not optimised for robust quantitative IVIM analysis, but in the healthy liver are adequate for assessing the pseudo-diffusion components of the proposed model (Jerome *et al* 2013). These data were then used to design Protocol 2 with a reduced set of *b*-value TE combinations added to a conventional multiple *b*-value diffusion protocol. A conventional 8-*b*-value acquisition (0, 10, 50, 100, 150, 200, 250, 400, and 800 mm^−2^s at TE 62 ms) was acquired with additional images taken at 3 *b*-values (0, 10, 50 mm^−2^s) with TE 80 and 100 ms. The overall acquisition time of Protocol 2 was approximately matched to Protocol 1 by increasing the number of signal averages, and Protocol 2 was evaluated on the remaining six volunteers. Volunteers imaged with Protocol 2 were scanned twice, approximately 1 month apart (median 29 days; range 14–43), in order to assess repeatability of the derived parameters. All imaging was performed in free-breathing using a 1.5 T MAGNETOM Avanto clinical MR scanner (Siemens Healthcare, Erlangen, Germany), using a prototype echo-planar imaging diffusion sequence that allowed explicit control of diffusion delay (*δ*) and diffusion time (Δ). DWI data were acquired coronally (to simplify subsequent image registration) using a 3-scan trace-weighted monopolar diffusion scheme, each scan stored separately, with TR  =  4000 ms (sufficient to avoid *T*_1_ weighting), FOV  =  380  ×  380 mm^2^, 16 contiguous 5 mm slices, matrix  =  128  ×  128 (interpolated to 256  ×  256), bandwidth  =  1628 Hz/pixel, spectral adiabatic inversion recovery (SPAIR) fat suppression, and generalised autocalibrating partially parallel acquisition acceleration (GRAPPA) factor 2. The TEs and *b*-value combinations, described above and represented schematically in figure 1, were acquired with the additional parameters:

Protocol 1: *δ*  =  17.6 ms, Δ  =  24.0 ms, 6/8 partial Fourier acquisition, and 5 repeated acquisitions. Thirty *b*-TE combinations were acquired in a regular array (*b*: 0–250 mm^−2^s, TE: 62–102 ms), for a total acquisition time of 36 min for each of *n*  =  5 volunteers.

Protocol 2: *δ*  =  16.0 ms, Δ  =  20.2 ms, 7/8 partial Fourier acquisition, and 12 repeated acquisitions. A reduced set of 14 *b*-TE combinations were acquired (see also figure 1) that includes an additional small *b*-value (*b*  =  10 mm^−2^s), which more closely matches typical clinical multi-*b*-value DWI protocols, giving a total acquisition time of 25 min for each of *n*  =  6 volunteers.

### 2.2. Data analysis

All analysis was performed using in-house software developed with MATLAB (The Mathworks Inc., Natick, MA USA). For each volunteer scan, a single slice showing the largest cross section area of the liver was chosen for analysis, without excluding large vessels or ducts; in repeated datasets, the matching slice was chosen. Images were acquired without averaging (Jerome *et al* 2014), and alignment of every image within each dataset (*b* and TE) was performed using a rigid shift based on the liver-diaphragm boundary, and a region of interest (ROI) was drawn covering the whole liver section.

For the exploratory data in Protocol 1, the signal intensity was averaged for all voxels in the ROI for each image, and this value was used in the model fitting. The extended T2-IVIM model was applied using all images, and in addition, the standard IVIM model was applied independently for all images at each TE. For Protocol 2, the T2-IVIM model was applied to all data, as well as the standard IVIM model for the conventional IVIM data (8 *b*-values) acquired at the lowest TE value (62 ms). Initial parameter values for IVIM fitting were obtained by a two-step fitting of a mono-exponential function to data points with *b*  >  150 mm^−2^s to provide an estimate of *D*. This fit is then projected back to *b*  =  0 mm^−2^s to provide an estimate of *f* from the excess measured signal at *b*  =  0 mm^−2^s value above the projected intercept, expressed as a fraction. For the T2-IVIM modelling, the same initialisation strategy can be used alongside an estimate of *T*_2*t*_ from fitting the multiple-TE data at *b*  =  50 mm^−2^s as this is sufficient to effectively remove the flow component in the liver (Jerome *et al* 2013).

For data from Protocol 2, both IVIM and T2-IVIM models were also fitted in a voxel-by-voxel basis to allow generation of functional parameter maps across the whole liver ROI. Lastly, in order to assess the use of the extended T2-IVIM model within a realistic clinical DWI examination time, a ‘minimised’ subset of the Protocol 2 data using only the first six repeated acquisitions of each *b*-TE combination, and excluding the scans at *b*  =  10 mm^−2^s for TE 80 and 100 ms (figure 1), for a total effective acquisition time of 10 min 45 s. The repeated measures coefficient of variation (CoV, %) was calculated for each parameter across the cohort in Protocol 2, using log-transformed measurement values (Limpert *et al* 2001). Statistical analysis of derived IVIM parameters was performed using analysis of variance (ANOVA) for repeated visits across the IVIM and T2-IVIM models.

## 3. Results

**Figure 2.**
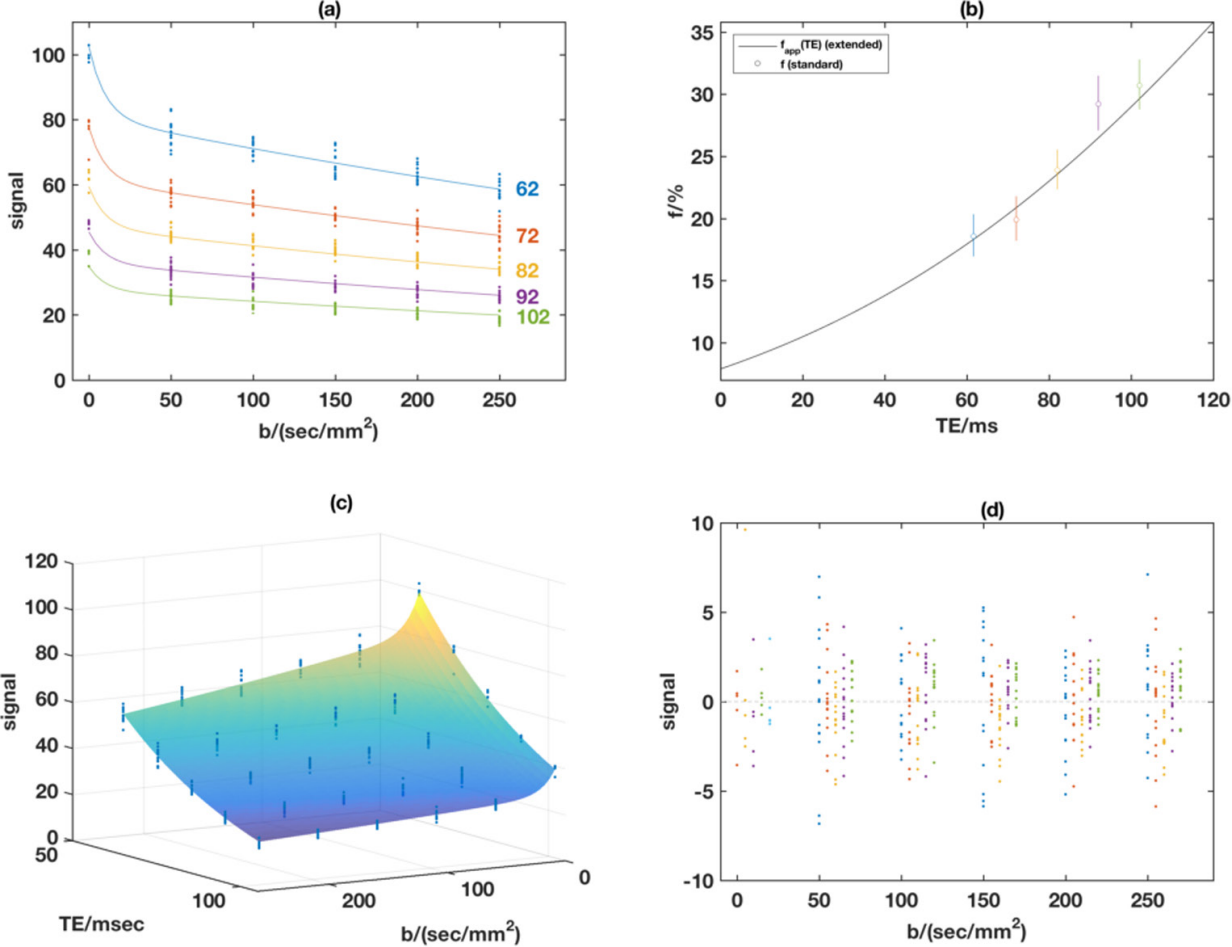
Example from volunteer in **Protocol 1.** Panel (a) shows the measured signals and fits using the standard IVIM for each TE separately; (b) shows the estimated *f* values from the standard fits in (a) with 95% standard error bars, and also the variation of the apparent pseudo-diffusion volume fraction (equation (3)) obtained using the parameter estimates with the extended model. Panel (c) shows the measured signals and fitted surface using the extended T2-IVIM model, with (d) showing the fit residuals from the T2-IVIM model for all combinations of TE and *b*-value using the same TE colour-coding as panel (a) with the smallest TE on the left of each group.

Figure 2 shows a typical example data set using Protocol 1, with the standard IVIM model applied at each TE (figure 2(a)); in figure 2(b), the TE-dependence of the pseudo-diffusion volume fraction computed using the standard IVIM model is shown (open circles) along with a curve showing the apparent pseudo-diffusion volume fraction (equation (3)) as a function of TE computed using parameters obtained from the extended T2-IVIM model fit. The limit of this curve at TE  =  0 ms gives the TE-independent pseudo-diffusion volume fraction, and the figure shows that this is significantly lower than that reported by the standard IVIM model at all echo times. Fitting the whole dataset with the extended T2-IVIM equation yields a surface that accounts for all the variation in the data (figure 2(c)), and this also shows the *T*_2_ attenuation with TE for all *b*-values. Figure 2(d) shows that there are no systematic features in the fit residuals.

Table 1 gives *f* estimates for each TE from Protocol 1 for each volunteer, and shows that the apparent increase in *f* at higher TE is consistent across the volunteer cohort. Acquiring multiple *b*-value images at a non-zero minimum TE consistently overestimates the pseudo-diffusion volume fraction, by a variable amount that reaches over 100% in three cases, and which may be expected to vary across scanners. The effect is exacerbated at longer TEs, indicating that the overestimation is dependent on the exact acquisition protocol.

**Table 1.** Apparent perfusion volume fraction *f* calculated from mean of ROI data from **Protocol 1** using conventional IVIM model (equation (1)) at each unique TE. There is an increasing overestimate of *f* with longer TEs across the cohort.

TE (ms)	Volunteer					Mean ± s.d.
	1	2	3	4	5	
102	0.305	0.227	0.474	0.209	0.322	0.307 ± 0.105
92	0.275	0.224	0.432	0.186	0.308	0.285 ± 0.094
82	0.246	0.222	0.391	0.165	0.295	0.264 ± 0.085
72	0.220	0.220	0.351	0.146	0.282	0.244 ± 0.077
62	0.195	0.217	0.311	0.128	0.269	0.224 ± 0.070
T2-IVIM model	0.090	0.203	0.136	0.057	0.199	0.137 ± 0.065
% error for TE = 62	117.7	6.9	128.9	126.0	35.1	82.9 ± 57.5

**Figure 3.**
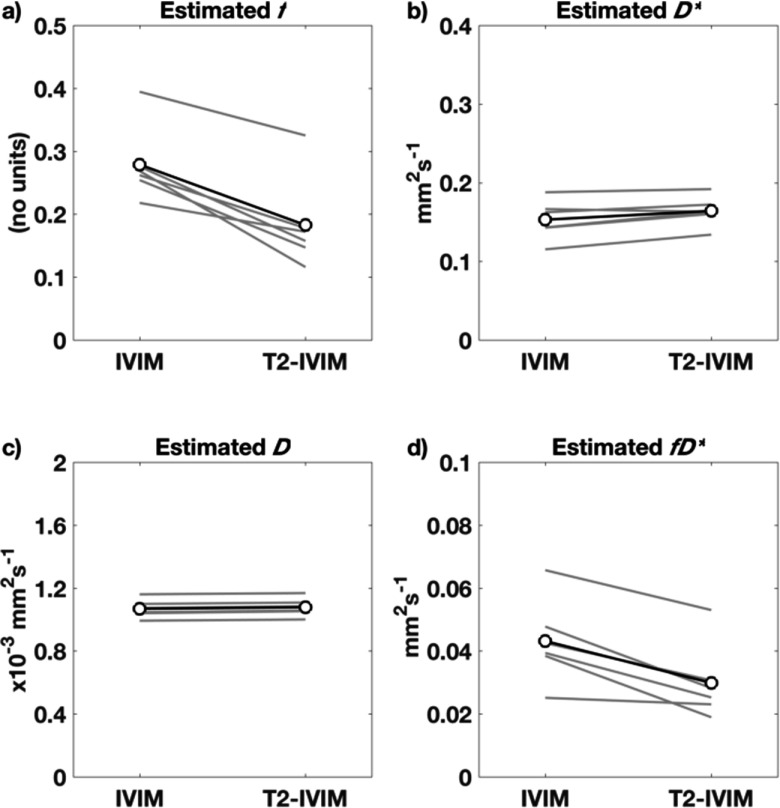
Ladder plots of *f*, D^*^, *D*, and fD^*^ for individuals (grey, average of repeat measures) and mean (black) across the cohort, fitting the mean ROI signal of data using **Protocol 2**.

Parameters derived from fitting the conventional IVIM and the extended T2-IVIM model using Protocol 2 are given in table 2, which reports both the mean and standard deviation for the liver ROI across the cohort, for the cases of fitting the ROI mean signal in each image, the ROI median value of the voxel-wise fitting, and the ROI mean signal from the ‘minimal’ subset of *b*-TE and signal averages. The median is less sensitive to outliers such as partial voluming of different tissues at the ROI edge, and large vascular features within the liver, and is commonly reported as a more robust summary statistic than the mean. No parameters were found to be significantly different between repeated scans, in either model (*p*  >  0.20 in all cases, ANOVA), or between the minimal or full data sets (*p*  >  0.70, ANOVA). The values for *D* and D^*^ were not significantly changed between the IVIM and T2-IVIM models (*p*  =  0.50 and 0.20 respectively, ANOVA), but were both lower when considering the median voxel value when compared to the mean (*p*  >  0.05, ANOVA); values for D^*^ are reported here as output of the fitting process, but owing to the known difficulty of obtaining accurate values for D^*^, interpretation of this parameter is explicitly avoided in this work. Values for *f* are significantly lower for the T2-IVIM model (*p*  <  0.0001, ANOVA). These results, also shown as ladder plots of the IVIM parameters (from fitting mean of ROI) in figure 3, clearly show that the effect of modelling for separate *T*_2_ consistently and significantly reduces the estimated value of *f*. The values for the compound parameter fD^*^ similarly drop when using the T2-IVIM model (*p*  <  0.001, ANOVA).

**Table 2.** Diffusion parameters from (i) mean of ROI signal, (ii) median of voxel- by-voxel fitting, and mean of ROI from ‘minimal’ sampling of **Procotol 2** data. Results are mean  ±  s.d. across the cohort, **Bold** indicates *p*  <  0.05 for *t*-test between IVIM and T2-IVIM models.

	ROI mean	Voxel-wise median	Minimal set
	IVIM		
*f*	0.279 ± 0.06	0.240 ± 0.06	0.277 ± 0.06
D^*^ (mm^2^ s^−1^)	0.153 ± 0.025	0.137 ± 0.025	0.161 ± 0.024
*D* (10^−3^ mm^2^ s^−1^)	1.07 ± 0.06	1.01 ± 0.06	1.08 ± 0.05
fD^*^ (mm^2^ s^−1^)	0.043 ± 0.013	0.033 ± 0.011	0.045 ± 0.012

	T2-IVIM		
*f*	**0.183 **±** 0.07**	**0.179 **±** 0.07**	**0.181 **±** 0.06**
D^*^ (mm^2^ s^−1^)	0.164 ± 0.019	0.148 ± 0.016	0.161 ± 0.025
*D* (10^−3^ mm^2^ s^−1^)	1.08 ± 0.06	1.03 ± 0.06	1.08 ± 0.05
fD^*^ (mm^2^ s^−1^)	0.030 ± 0.013	0.026 ± 0.011	0.029 ± 0.011
*T*_2*p*_ (ms)	77.6 ± 30.2	82.0 ± 44.5	90.9 ± 47.7
*T*_2*t*_ (ms)	42.1 ± 6.8	41.5 ± 6.5	42.3 ± 6.4

**Figure 4.**
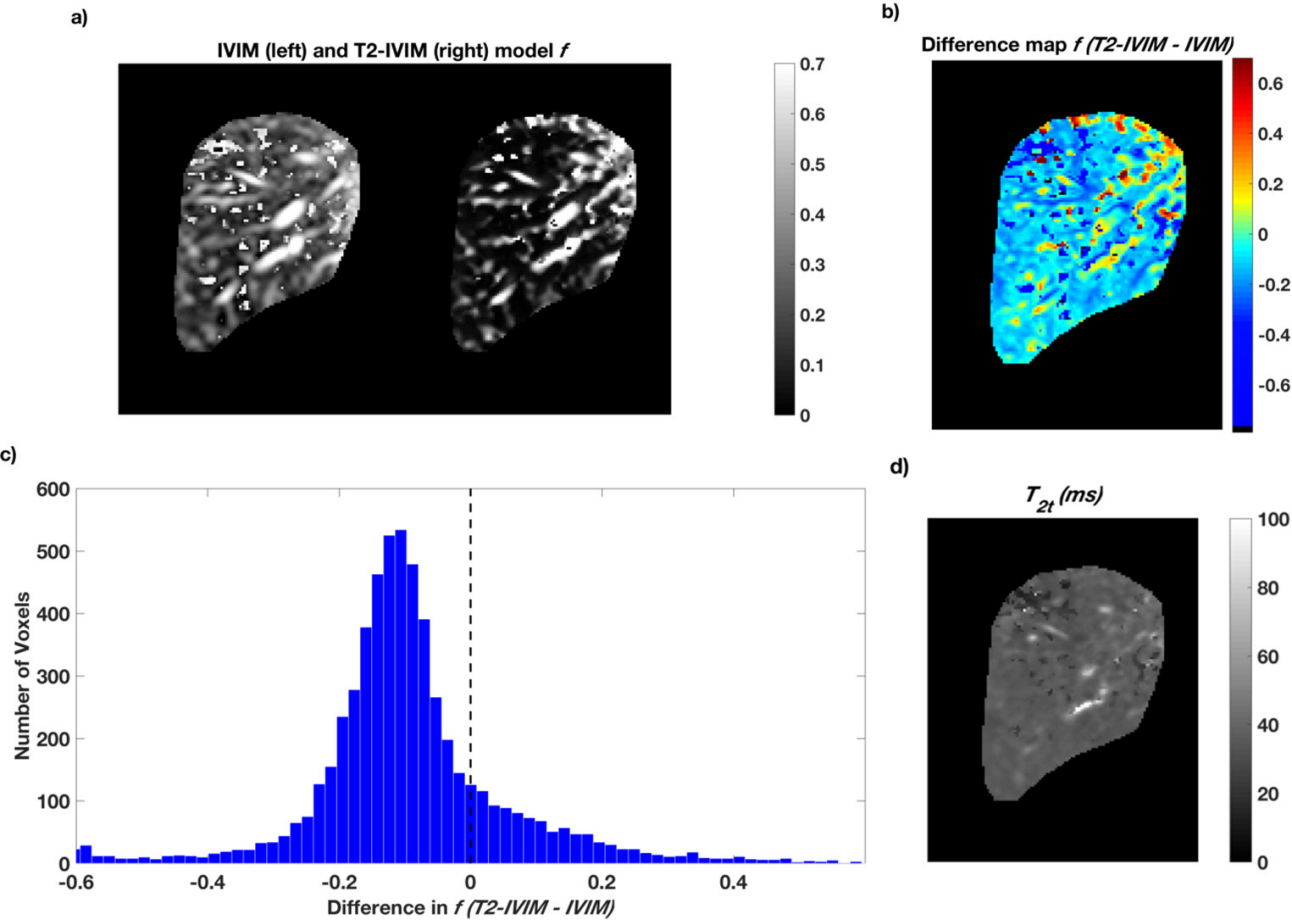
(a) Calculated pseudo-diffusion fraction *f* maps from IVIM (left) and T2-IVIM (right) models using Protocol 2 data. The difference map (b) and histogram (c) show the reduction in *f*, given by T2-IVIM value minus IVIM value, observed when allowing for different *T*_2_*s* in the model compartments. The true-diffusion compartment *T*_2_ map is shown in (d).

A typical example of *f* maps from the two models, from voxel-by-voxel model fitting, is presented in figure 4(a); the smaller *f* observed in the T2-IVIM fitting is consistent in the liver parenchyma and well-visualised as a difference map, and by the histogram of *f* differences (figures 4(b) and (c)). Vascular features are retained in the T2-IVIM *f* map, showing the ability of the model to deal with varying tissue composition through the ROI. Voxels where the estimate of *f* is higher in the T2-IVIM model appear to correspond to large vascular features within the liver, and at the top boundary of the liver where there may be increased uncertainty owing to respiratory motion. The difference map also shows that over smaller ROIs in the liver parenchyma, such as may correspond to the size of a lesion, the reduction in *f* is consistently present.

Estimation of the *T*_2_ values for the pseudo- and true diffusion compartments derived from the T2-IVIM model, given in table 3, show good repeatability for *T*_2*t*_ (CoV  <  10%) but less so for *T*_2*p*_, with correspondingly high variation (CoV  >  20%) seen for *T*_2*p*_ and *f*. The values given for *T*_2*t*_ are consistent with literature values (Stanisz *et al* 2005), although the *T*_2*p*_ is lower than literature values for blood at 1.5 T. A representative *T*_2*t*_ map from the T2-IVIM model is shown in figure 4(d), and is largely free of vascular features.

**Table 3.** Coefficients of variation (%) of parameters derived from conventional IVIM and T2-IVIM modelling of data from **Protocol 2**, for fitting mean of ROI, median of voxel-by-voxel, and using the minimally-sampled dataset.

	ROI mean	Voxel-wise median	Minimal set
	IVIM		
*f*	4.9	7.0	6.3
D^*^	6.1	9.6	12.6
*D*	5.0	4.9	4.5
fD^*^	7.7	10.7	13.6

	T2-IVIM		
*f*	23.4	35.0	30.5
D^*^	4.3	5.6	14.7
*D*	4.5	4.5	4.5
fD^*^	24.4	36.7	37.3
*T*_2*p*_	21.4	25.4	33.8
*T*_2*t*_	6.3	7.0	6.7

**Figure 5.**
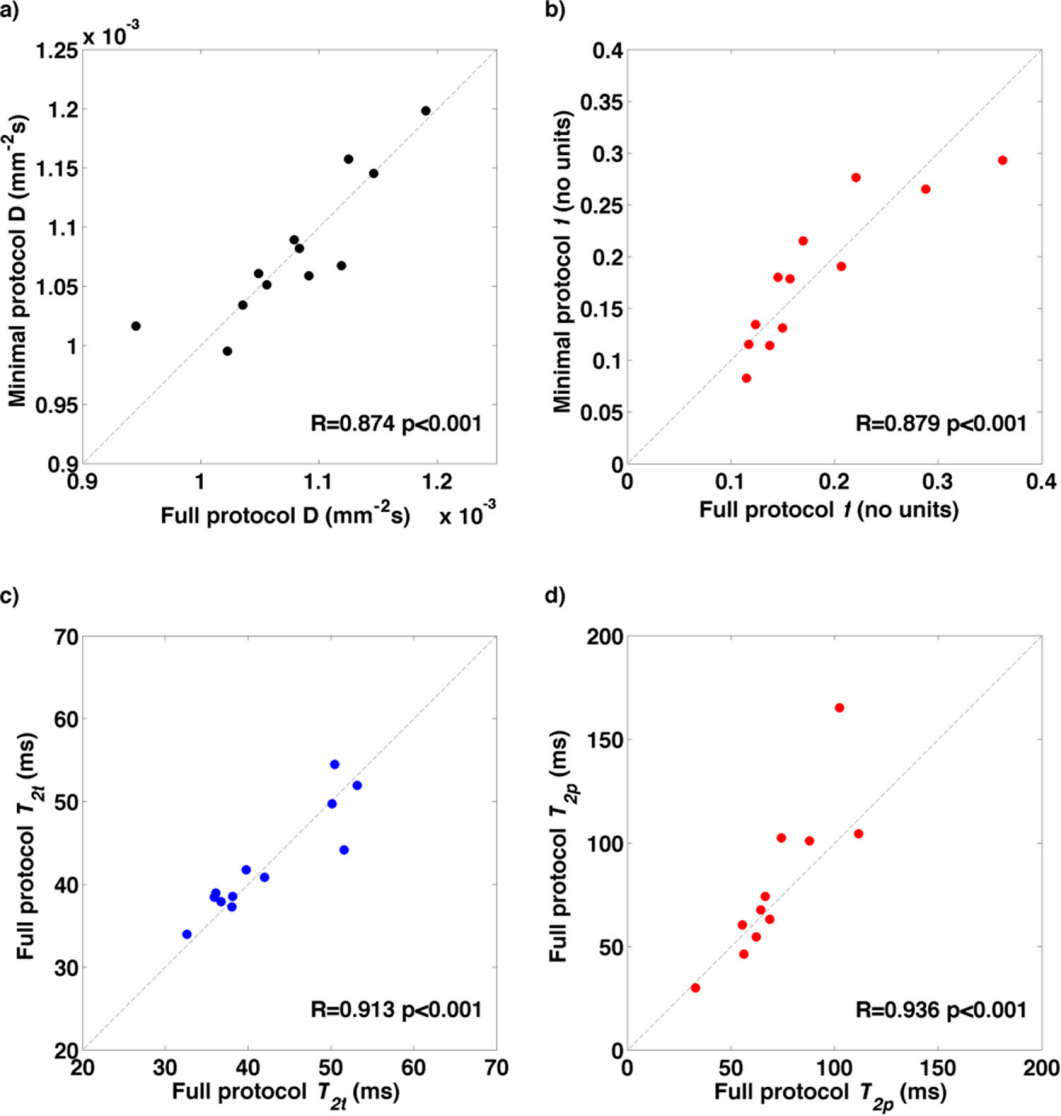
Correlation of T2-IVIM parameters for fitting the mean ROI signal between the full **Protocol 2** and the minimal set acquired within a clinical time frame: (a) *D* (×10^−3^ mm^2^ s^−1^), (b) *f* (%), (c) *T*_*2t*_ (ms), and (d) *T*_*2p*_ (ms).

The same parameters derived from the ‘minimal’ subset of data from Protocol 2, limited to simulate the constraints of acquisition within a clinical time frame, show results for the T2-IVIM fitting that are equivalent to those derived from the full dataset (figure 5). The CoVs for the minimal dataset also match those from the full set; namely, below 10% for *D* and *T*_2*t*_, but higher from parameters relating to the pseudo-diffusion volume fraction (*f*, D^*^, and *T*_2*p*_).

## 4. Discussion

More complex diffusion models than assumed monoexponential decay have the potential to inform on microstructure in tissues where more complex phenomena than Gaussian diffusion are present. In many tissues, a second exponential is included, giving the IVIM model, which allows inference of vascular fraction and tissue perfusion properties. Such measures are becoming increasingly important in oncology to differentiate tumour type or grade (Yamada *et al* 1999, Chandarana *et al* 2011), or where the focus of a treatment may be anti-angiogenic or reducing tumour vascularity (Kim *et al* 2016, Yang *et al* 2016). In using models with additional parameters, however, there is the potential for over-fitting, or having strongly covariant parameters that do not supply additional information. Conversely, if the model being used to fit the data is an insufficient description of the system being studied, unmodelled variation in the data will manifest as bias in the parameter estimates, and confound any conclusions. In addition, any derived parameter being used as a biomarker, e.g. for diagnosis or treatment response, must be understood in terms of its sensitivity, reproducibility across scanners/sites, and stability across repeated measurements.

The parameter *f*, derived from the IVIM model, is formulated and often reported as the perfusion or vascular fraction; while the original discussion of the model identified the compound parameter fD^*^ as related to perfusion (Le Bihan *et al* 1988), this work also identified that failure to allow for distinct *T*_2_ values for the two components in the IVIM model results in *f* having a TE dependence. In cases where the two compartments have sufficiently different *T*_2_ values, the estimation of *f* will be significantly affected. In practical terms, the minimum TE often used for clinical DWI is determined by the diffusion encoding scheme, the gradient hardware, and the maximum *b*-value used; it is thus problematic to draw general conclusions regarding *f* where the data may have been acquired using different TEs. This sequence variation is common for acquisitions in different centres where different scanners are used, and so this issue will be particularly relevant for multicentre clinical trials where rarity of disease mandates participation from many sites. A TE-independent *f* would remove this problem and potentially increase the utility and specificity of *f* (and fD^*^), especially in the multicentre trial setting as relating to perfusion.

In this study we show that a complete formulation of the IVIM model, extended to allow for distinct *T*_2_ values in the two diffusion compartments, can account for the observed variation in *f* with TE. Extending data acquisition to include multiple TE as well as *b*-values, combined with fitting the T2-IVIM model allows estimation of compartment *T*_2_*s* alongside conventional IVIM parameters. We demonstrate that this new T2-IVIM model can be applied in a clinically relevant setting, adding only a small number of scans to a conventional multiple-*b*-value DWI protocol. With current interest in optimising *b*-values for IVIM acquisition (Jambor *et al* 2014, Kaya and Koc 2014, Leporq *et al* 2015), addition of *b*-values at distinct TEs is entirely feasible for routine clinical studies, and will provide facility for T2-IVIM modelling as well as greater data support for estimation of *S*_0_, crucial for the IVIM model.

The results of this study, consistent with previous work (Lemke *et al* 2010), show that with a finite and non-zero TE, *f* in the normal liver is routinely overestimated; with a typical TE of 62 ms, such overestimation appears variable across subjects, but in several cases is over 100%, which indicates that *f* cannot be straightforwardly interpreted as a perfusion volume fraction. If the overestimation is due to a differential *T*_2_ in the pseudo-diffusion compartment, then oxygenation status of the blood may also play a role in the *f* value derived, again suggesting that *f* cannot be reliably interpreted as vascular fraction. Certainly, the use of different TEs will create variation in observed *f*, and so the use of the extended T2-IVIM model may thus lead to a greater ability to standardise the use of IVIM for clinical trials, particularly in a multi-centre setting or where repeated MR scans are performed on different scanners.

Previous work by Lemke *et al* using a literature value for tissue and blood promises retroactive compensation for overestimation of *f*, but this is based on the dual assumptions that the literature *T*_2_ value is correct, and that the IVIM model itself correctly identifies the pseudo-diffusion volume fraction as pure blood. The T2-IVIM method avoids these assumptions, and directly estimates the separate *T*_2_ values; our results in this study consistently show a *T*_2_ of the pseudo-diffusion compartment in the healthy liver of around 80 ms, which is notably different to the literature value of 290 ms for blood used by Lemke *et al*, and suggests that the use of a literature value, while having the benefit of being applicable to legacy data following acquisition, may give a significant underestimation of *f* and thus be an imperfect or partial solution compared to allowing the *T*_2_ value to be estimated from prospectively acquired data. This discrepancy in derived pseudodiffusion compartment *T*_2_ may highlight the difficulty of measuring blood *T*_2_ *in vivo*, or may reflect phenomena such as water exchange unaccounted for in the IVIM model. The *T*_2_ values found for the liver tissue in this study are consistent with literature values (Stanisz *et al* 2005).

Repeatability estimates found for IVIM parameters in this work are remarkably good, with small coefficients of variation (<10%) for *f*, D^*^, and *D* using the standard IVIM model in the liver. While *D* has previously been found to be highly repeatable, the pseudo-diffusion parameters are commonly reported to be less stable in tumours (Jerome *et al* 2016), thus caution is required when using these parameters to infer physiological change (Winfield *et al* 2015). For fitting with the extended T2-IVIM model, CoV values for *D* and D^*^ remain low, and there is a correspondingly low CoV for the *T*_2_ of the slow diffusion compartment (6.3%). The CoVs for *f* and *T*_2*p*_ are larger, at  >20%, and reflect how variations in *T*_2*p*_ and *f* manifest the same way in the diffusion decay curve. The small reduction in CoV observed for D^*^ may indicate an improved ability of the T2-IVIM model to separate *f* and D^*^, and the low CoV of the *T*_2*t*_ parameter suggests that it may be used as a robust imaging marker in a more specific sense than the (more commonly used) apparent *T*_2_ (Raza *et al* 2012, Guimaraes *et al* 2016).

It is worth noting that in diagnostic applications where the comparison between normal and pathological *f* values is of primary interest, the superior repeatability of the conventional IVIM model may be advantageous. However, in applications where longitudinal changes occur, where there is comparison between individuals, or in the context of a multi-centre clinical trial, the additional information provided by the T2-IVIM may outweigh the increased CoV for this model.

Most importantly for this study, the increased CoV observed for *f* in the T2-IVIM model compared to the conventional IVIM model, highlights the strength of the interdependence of *f* and *T*_2*p*_, and indicates that the apparent robustness of *f* estimates from the conventional IVIM model may be superficial. These results indicate that a greater investment of scanning time into *T*_2_ estimation, from either an extended TE range or greater TE sampling, would help to reduce the CoV on *T*_2*p*_ and by extension *f*. The CoV for *f* from T2-IVIM in this study is still lower than that seen for *f* in many IVIM repeatability studies in tumours (Dyvorne *et al* 2014, Winfield *et al* 2015, Jerome *et al* 2016), underscoring the difficulty of interpreting these parameters in the clinical context. In single-scanner settings where the scanning protocol is unchanged, these data show that whilst the conventional IVIM scheme gives lower CoV than the T2-IVIM scheme, for this to translate into improved treatment sensitivity it is necessary to assume that the treatment does not change the *T*_2_ of blood in the pathology. The relative simplicity and robustness of the conventional IVIM approach lends itself to diagnostic utility, but the T2-IVIM scheme has the potential to provide data in a more controlled setting to further our understanding of a potentially important source of variation affecting *f* estimates in different pathologies. In addition, accounting for known influence of acquisition parameters will increase the reliability of cross-scanner comparisons.

Limitations of this study include the lack of an available gold standard to measure the actual vascular fraction, since this appeared to be variable across the volunteers, and the limited range of TE used for *T*_2_ estimation due to limitations in available SNR. Furthermore, we have not applied this technique to evaluate a range of oncological pathologies, which will be pursued in prospective studies. In this study, the effects of diffusion time and gradient profile were not explored, which may be expected to have an effect on the estimated *f* (Thian *et al* 2014).

In conclusion, the standard IVIM model is liable to give inaccurate values for the pseudo-diffusion volume fraction, often termed the perfusion fraction, owing to the invalid assumption that the two model compartments have similar *T*_2_ relaxation. The larger *T*_2_ value in the pseudo-diffusion volume fraction will lead to a consistent overestimation of *f*, which will be dependent on the intrinsic characteristics of the spins in that compartment, and is not simply the perfusion fractional volume. Acceptable CoV for such parameters may give false confidence in their accuracy or utility of such, and is a serious confounding factor for IVIM modelling. The addition of two low *b*-value scans at additional TEs allows estimation of the *T*_2_ values for each compartment alongside the standard IVIM parameters using the extended model, giving a more accurate picture of the pseudo-diffusion volume fraction, as well as the limit of useful interpretation, for use in clinical trials and improved standardisation of advanced diffusion model parameters.
